# Regulation of indigoidine production in *Vogesella indigofera* by a novel two-component system

**DOI:** 10.1128/jb.00364-25

**Published:** 2025-12-03

**Authors:** Madison Babcock, Kristin Romanelli, Samantha Gonzalez, Genevieve Robinson, Peter D. Newell

**Affiliations:** 1 Department of Biological Sciences, State University of New York at Oswego 14828, Oswego, New York, USA; National Institutes of Health, Bethesda, Maryland, USA

**Keywords:** pigment, regulation, *Vogesella*, signaling, biosynthesis

## Abstract

Indigoidine is a blue pigment synthesized by several bacteria, including *Vogesella indigofera*. Industrial production of indigoidine has been a research focus, but less is known about why bacteria make this pigment or how its biosynthesis is regulated. We isolated *V. indigofera* strain OSW_575 and investigated the basis for its indigoidine production using genomic and genetic approaches. Mutation of the indigoidine synthase gene *igiD* eliminated pigment production, and complementation restored it. A transposon mutagenesis screen uncovered 34 mutations across 20 genes that affect pigment production, including some involved in metabolism, translation, protein homeostasis, and regulation. Three chaperones that combat misfolded proteins, *dnaK*, *dnaJ*, and *grpE*, were required for indigoidine production, while mutations affecting the *clpAP* proteasome resulted in hyperpigmentation. These results are consistent with prior studies and suggest a role for the protein homeostasis system in regulating indigoidine. We also found that the alternative sigma factor *rpoN* contributes to indigoidine production. Finally, one transposon mutation affected a predicted sensor histidine kinase, which we dub *tciK*. Our genetic characterization of *tciK* and its cognate response regulator *tciR* suggests that they function in the same pathway to regulate indigoidine. TciR is of interest due to its non-canonical domain architecture that combines an N-terminal REC domain and C-terminal RsbW-like anti-sigma factor domain. Further investigation of this system may reveal novel regulatory mechanisms. To our knowledge, this is the first study to employ genetic tools in *V. indigofera*, and we propose it as a useful experimental system for studying the regulation and function of indigoidine.

**IMPORTANCE:**

Despite being known to science for more than a century, *Vogesella indigofera* has been the focus of few studies. We isolated a strain of this bacterium, sequenced its genome, and investigated which genes contribute to its production of the blue pigment indigoidine. We found that mutations in genes involved in metabolism, protein homeostasis, and regulation can affect pigment production. One locus required for indigoidine production encodes a novel two-component regulation system. We conducted a preliminary genetic characterization of this system, which includes a non-canonical response regulator. Based on the results, we propose our strain as a model organism for studying indigoidine production and regulation.

## INTRODUCTION

*Vogesella indigofera* was first isolated by Voges in 1893 (1), then later formally described by Grimes et al. as a betaproteobacterium that produces the pigment indigoidine (2). Betaproteobacteria are common constituents of freshwater bacterial communities. *Vogesella* has been proposed as a potential indicator of anthropogenic disturbance in freshwater systems, particularly nitrate contamination (3–5). Despite its early discovery and wide distribution, very few studies have focused on *V. indigofera*, and none have utilized genetic tools for its characterization.

Indigoidine is formed by a single-module non-ribosomal peptide synthetase from two l-glutamine molecules (6, 7). The metallic blue pigment is not water-soluble in its oxidized state, but can be reduced to leucoindigoidine, which is colorless and water-soluble (8). Indigoidine production has been reported in a diverse array of bacteria including Alpha-, Beta-, and Gammaproteobacteria, as well as several Actinobacteria, suggesting the gene encoding the synthase has been horizontally transferred (9). Most research on the pigment has focused on optimizing its production in heterologous hosts for industrial applications, especially as an alternative to synthetic dyes for textiles (6).

The biological role of indigoidine has been studied in relatively few organisms. For the phytopathogen *Dickeya dadantii*, it was shown to contribute to pathogenesis and provide resistance to reactive oxygen species (10). In *Vogesella* sp. EB, indigoidine and a derivative pigment, cryoindigoidine, have been proposed to act as cryoprotectants, as they improve survival after freezing (11). Indigoidine regulation and function has been best studied in the marine Roseobacter *Phaeobacter* sp. Strain Y4I by Buchan et al., who found that it can act as an antimicrobial (8). Producing indigoidine gives *Phaeobacter* a competitive advantage in mixed-species biofilms (12). A transposon mutation in the indigoidine synthase gene *igiD* had pleiotropic effects on the bacteria, reducing motility and increasing surface attachment. Interestingly, loss of indigoidine elevated hydrogen peroxide resistance in *Phaeobacter*, contrary to what was observed in *D. dadantii* (8). Altogether, it seems likely that the function of indigoidine varies depending on the organism and situation in which it is produced. Thus, much remains to be learned about the significance of indigoidine production by various bacteria in natural environments.

In many bacteria, including *D. dadantii*, *Photorhabdus luminescens*, and *Streptomyces lavendulae*, indigoidine production is not observed in wild-type (WT) strains under standard laboratory conditions (7, 9, 10), indicating that its expression is subject to regulation. In *Vogesella,* indigoidine production has been shown to be inhibited by hexavalent chromium (13) and induced by cold temperatures and growth on surfaces (11). *Phaeobacter* also produces more indigoidine when grown on surfaces at high density, and two distinct quorum-sensing systems were shown to control its expression (14). The authors of the latter study found that *igiD* transcript abundance increased prior to pigment production, and then the two metrics followed a similar, increasing trend over time. The molecular mechanisms regulating indigoidine production in *Vogesella* are unknown and have yet to be investigated.

We isolated a bacterium that produces a large amount of indigoidine when grown on plates. The goal of this study was to characterize the isolate and investigate the basis of its indigoidine production using genomic and genetic approaches. We identified the organism as *V. indigofera* through genome sequencing and multi-locus phylogenetics and identified a range of genes that impact indigoidine production in a transposon mutagenesis screen. We present this system as a new model for studying the production and regulation of indigoidine.

## RESULTS

We performed spread plating using the effluent of a stormwater drain on the shore of Lake Ontario as part of an education and outreach exercise. Among several hundred colonies observed was a single isolate that produced blue pigment. We found this organism to be a motile, Gram-negative, rod-shaped bacterium. Given the interest in indigoidine production for textiles and other uses (15), we chose to isolate and characterize this bacterium. Our objectives included identifying the species, sequencing its genome, and determining which genes contribute to the production and regulation of the pigment.

We obtained 3.54M paired, 150 bp Illumina reads of the isolate’s genomic DNA and assembled them using SPAdes (16). This produced a genome 3,494,071 bp in length, comprised of 23 contigs, with an *N*_50_ of 276,544 bp, an average coverage of 170×, and GC content of 64.45%. Genome quality was assessed with CheckM (17), and found to be 99.15% complete with no detectable contamination. A blastn search using the 16S rRNA gene sequence provided a preliminary taxonomic assignment, with our isolate’s sequence matching *V. indigofera* DSM 3303 with 98.93% identity. Next, we conducted genome-wide comparisons of our strain to the seven available *V. indigofera* genome sequences, as well as three other closely related *Vogesella* genomes. We used FastANI to compare nucleotide sequences (18), ezAAI to compare amino acid sequences (19), and the Type Strain Genome Server to perform digital DNA-DNA hybridization (dDDH) (20). This showed our strain shares 95.42-96.98% nucleotide identity (ANI) with *V. indigofera* representatives, and 93.5% ANI with the closely related congeneric *V. fluminis* KCTC 23713 (Table S1). The accepted ANI threshold for species assignment is 95–96% (18), leading us to provisionally name our isolate *V. indigofera* OSW_575. Average amino acid identity (AAI) comparisons showed a similar trend with >97% AAI in all pairwise comparisons among *V. indigofera* strains and <95% with *V. fluminis* (Table S2). However, dDDH comparisons to the type strain DSM 3303 for OSW_575 and four other *V. indigofera* genomes recently published by Lu et al. (21) showed values around 60% (Table S3), which is below the accepted 70% threshold for species assignment (20). In contrast, comparisons of DSM 3303 and *V. indigofera* strains LYT24W and SH7W, as well as *Vogesella* sp. AC12 and EB gave dDDH of 74–75% (Table S3), providing support for the inclusion of the latter two strains in the species *indigofera*. These results are discussed further below.

**Fig 1:**
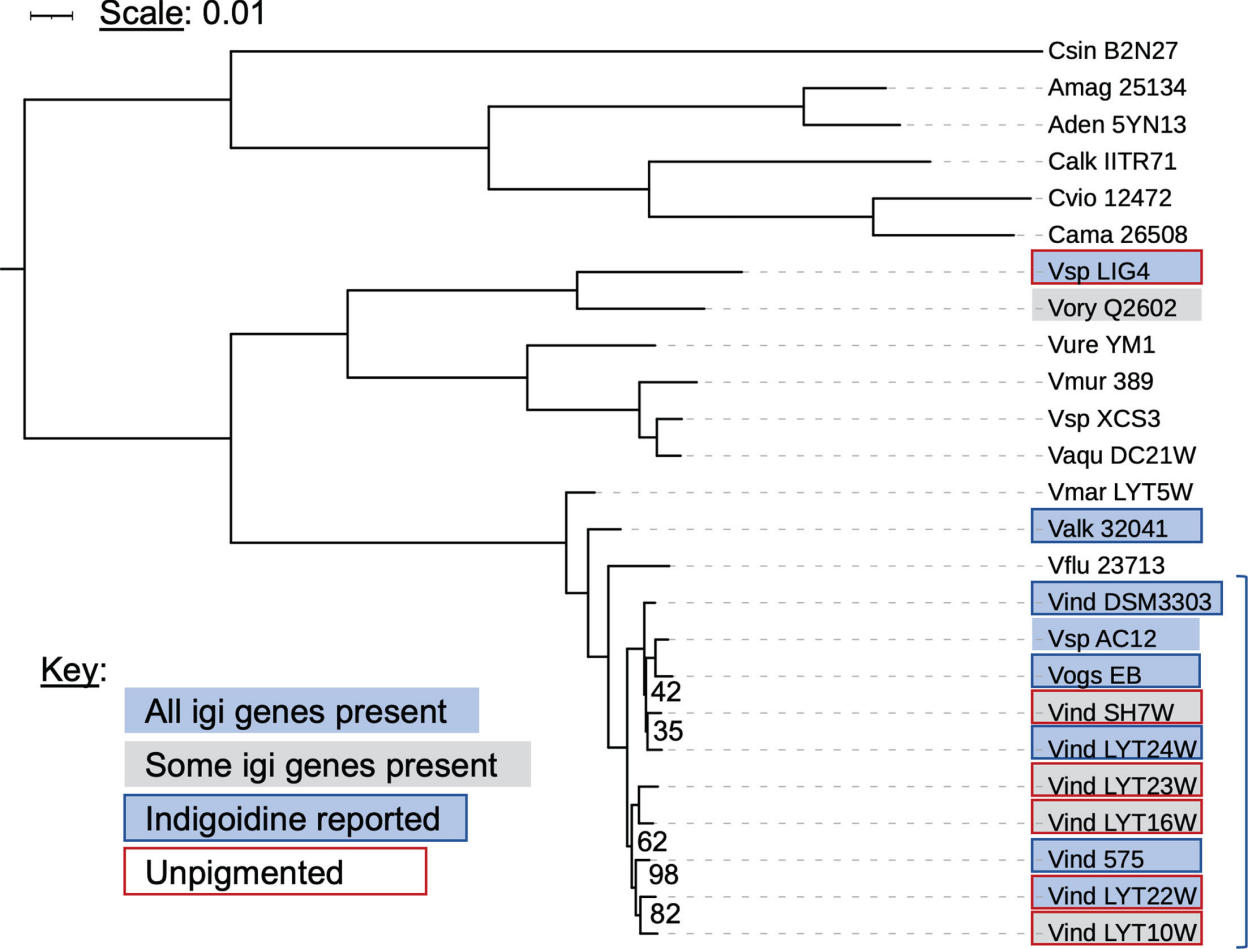
Phylogenetic analysis of *V. indigofera* OSW_575. A multi-locus phylogeny was constructed based on 93 single-copy, conserved proteins from the strains shown. The tree was built using iqtree2 with 100 bootstraps. Bootstrap values for all nodes were 100 unless otherwise shown. Strains of *V. indigofera* are indicated with a blue bracket on the right. A blue box indicates the presence of indigoidine biosynthetic genes *igiABCDEFG* in the strain. A gray box indicates *igiA-D* are absent. If the box is outlined in blue, blue pigment production has been reported for that strain, while a red outline indicates that the strain was described as unpigmented. Strains analyzed from top to bottom: *Craterilacuibacter sinensis* B2N2-7, *Aquitalea magnusonii* DSM 25134, *Aquitalea denitrificans* 5YN1-3; *Chromobacterium* species: *C. alkanivorans* IITR-71, *C. violaceum* ATCC 12472, *C. amazonense* DSM 26508; *Vogesella* species: *Vogesella* sp. LIG4, *V. oryzae* Q2602, *V. urethralis* YM-1, *V. mureinivorans* 389, *Vogesella* sp. XCS3, *V. aquatica* DC21W, *V. margarita* LYT5W, *V. alkaliphila* KCTC 32041, *V. fluminis* KCTC 23713; *V. indigofera* strains: DSM 3303, *Vogesella* sp. AC12, *Vogesella* sp. EB, *V. indigofera* SH7W, *V. indigofera* LYT24W, *V. indigofera* LYT23W, *V. indigofera* LYT16W, *V. indigofera* OSW_575, *V. indigofera* LYT22W, and *V. indigofera* LYT10W.

To clarify the phylogenetic relationships between *V. indigofera* OSW_575 and other indigoidine-producing relatives, we built a multi-locus phylogeny using sequences of 93 single-copy marker protein sequences from the genomes of 24 related bacteria (Fig. 1). Concurrently, we used a pangenome built with OrthoMCL to determine which species contain orthologs of the indigoidine synthesis genes, encoded in the *igi* locus described in *Vogesella* (11). The phylogeny places our isolate with other published *V. indigofera* species, with strong bootstrap support (100) for their separation from *V. fluminis* and other *Vogesella* species (Fig. 1). *V. indigofera* strains form two distinct clades, with LYT24W, SH7W, *Vogesella* sp. AC12 and EB all clustering with DSM 3303 and the remainder grouping with OSW_575. The separation of these groups was supported by 100 bootstraps and follows the trend observed in the dDDH data (Table S3).

Interestingly, we found that four strains of *V. indigofera* described as unpigmented by Lu et al. (21) lack the *igi* locus including the synthase gene *igiD* (Fig. 1). Most strains with a full complement of *igi* genes have been reported to produce indigoidine, with the notable exception of *V. indigofera* LYT22W and *Vogesella* sp. LIG4, which contains the locus but was reported to be unpigmented (21, 22). This speaks to potential differences in *igi* gene regulation among *Vogesella* strains and is reminiscent of prior descriptions of indigoidine production as a “cryptic” trait of some bacteria that only express it under certain conditions (9).

### *igiD* is required for pigment production by *V. indigofera* OSW_575

**Fig 2:**
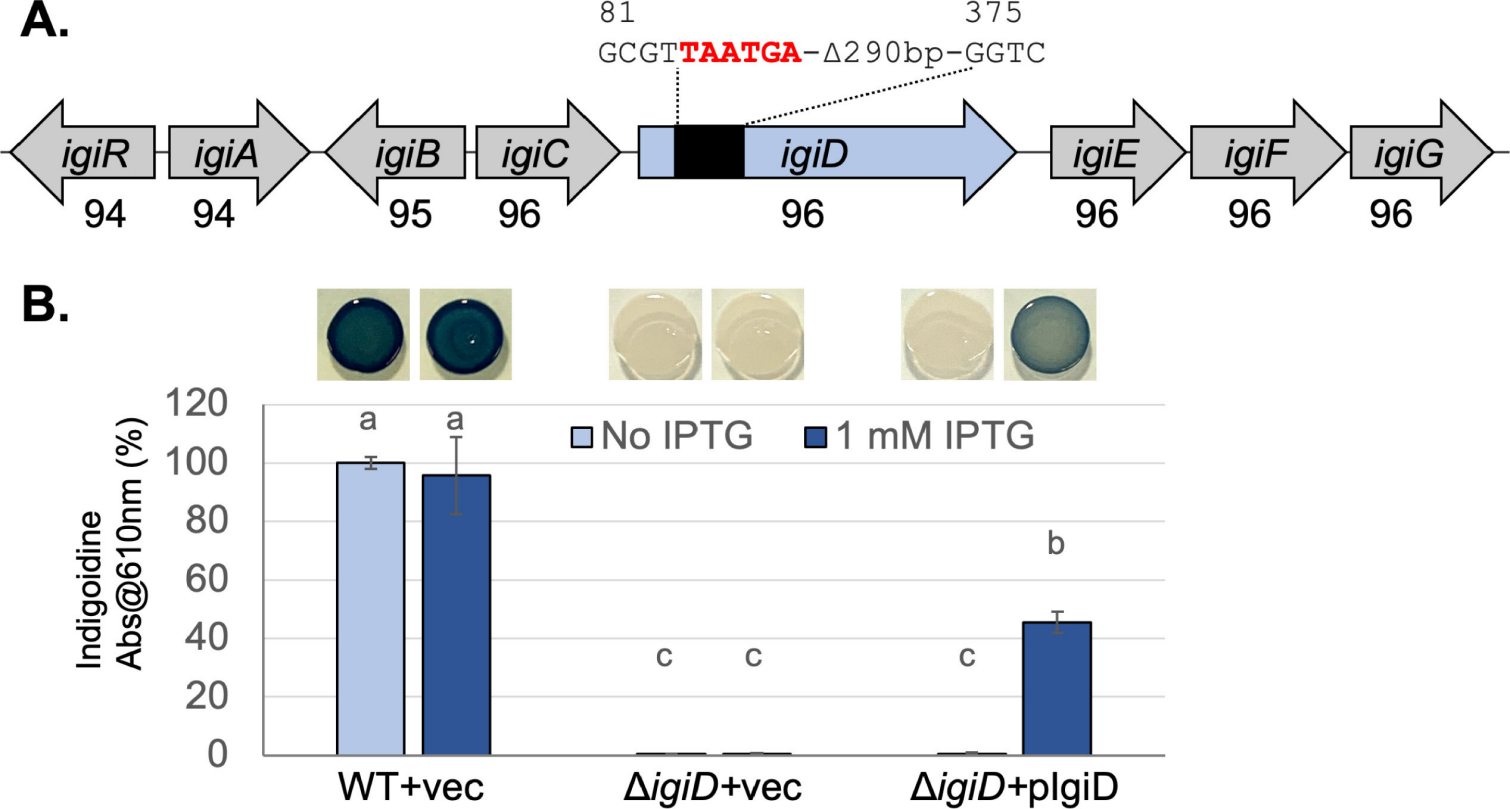
Indigoidine biosynthetic genes in *V. indigofera* OSW_575. (**A**) The indigoidine biosynthetic locus is shown. Numbers below the genes signify the percent nucleotide identity to genes in *V. indigofera* DSM 3303, determined by blastn. Allelic exchange was used to modify the indigoidine synthase gene, *igiD*, as shown above, with numbers indicating the nucleotide position relative to the start of the reading frame and stop codons added to the reading frame shown in red. (**B**) Indigoidine was solubilized and quantified as absorbance at 610 nm, from strains grown under conditions with and without IPTG, shown as a percent of WT in the No IPTG condition (mean ± standard deviation). Strains labeled + vec contain pSRKKm, while Δ*igiD* + pIgiD contains the plasmid with igiD under IPTG-inducible control. Different lowercase letters above the bars indicate *P* < 0.05 in pairwise *t* tests after Bonferroni correction; *n* = 6 from three independent experiments. Representative images of spot cultures growing on TSA plates with or without IPTG are shown above the corresponding bars.

To test the role of *igiD* in pigment production, we used allelic exchange to introduce a deletion mutation near the beginning of the reading frame (Fig. 2A). To test for complementation, we cloned the *igiD* gene into the broad host-range plasmid pSRKKm downstream of the isopropyl β-d-1-thiogalactopyranoside (IPTG)-inducible promoter (23), and moved the plasmid into the mutant via conjugation. We found that mutation of *igiD* eliminated pigment production, while reintroduction of the gene on the plasmid partially restored it when IPTG was added (Fig. 2B). The presence of IPTG and/or the empty vector did not affect pigment production by the mutant or WT. These data are consistent with *igiD* encoding an indigoidine synthase that is required for *V. indigofera* OSW_575 pigment production.

### Transposon mutagenesis screen

**Fig 3:**
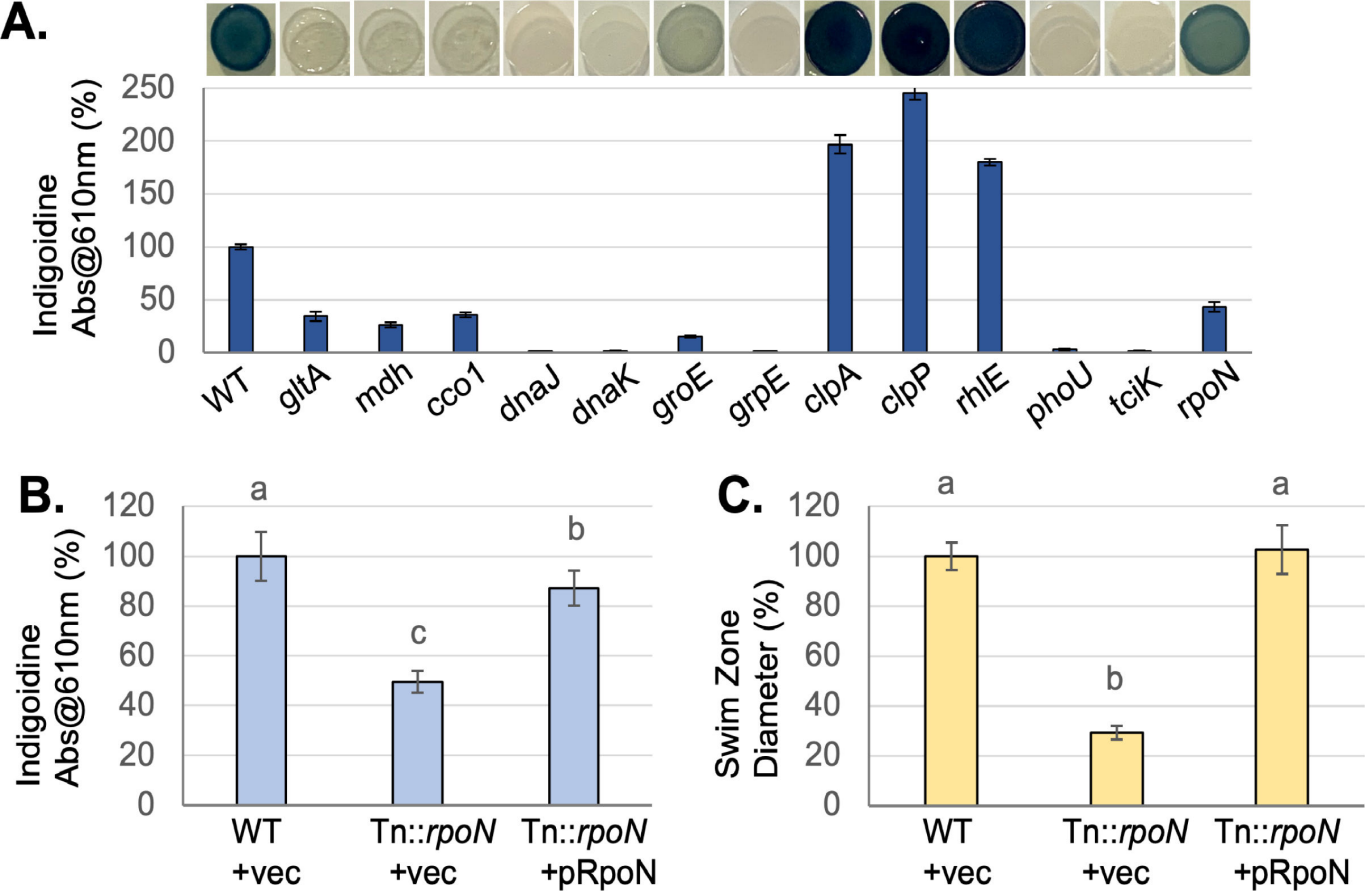
Phenotypic characterization of transposon mutants. (**A**) Indigoidine production, quantified as absorbance at 610 nm, is shown as a percent of WT (mean ± standard deviation) for selected mutants from Table 2, *n* = 6. Representative images of spot cultures growing on TSA plates are shown above the bars. (**B**) Indigoidine production is shown as a percent of WT (mean ± standard deviation). Strains labeled + vec contain pOSW4, while pRpoN contains pOSW4 with the *rpoN* gene. Different lowercase letters above the bars indicate *P* < 0.05 in pairwise *t*-tests after Bonferroni correction; *n* = 9 from three independent experiments. (**C**) Motility was quantified by measuring swim zone diameter in soft agar plates, shown as a percent of WT for the strains in (**B**), *n* = 9 from three experiments (mean ± standard deviation).

Consistent with observations in other bacteria, *V. indigofera* OSW_575 produces indigoidine when growing on plates but not in shaking culture, demonstrating some level of regulation. Quorum-sensing systems regulate indigoidine production in *Phaeobacter* (14), but blast and annotation searches of the *V. indigofera* OSW_575 genome found no evidence for quorum-sensing genes. To expand our knowledge about indigoidine regulation in *Vogesella*, we implemented a genetic screen for transposon mutants with altered pigment production. Approximately 15,000–20,000 Mutant colonies were screened visually for increased or decreased pigment production, and transposon insertions were sequenced and mapped. Representative data are pictured in Fig. 3A, and the full list of mutants is in Table 1. Assuming random insertion of Tn5, and an average gene length of 790 bp (calculated from our genome sequence), we estimated a 96% saturation of the genome by the method of Zhang et al. (24).

**TABLE 1:** Transposon mutants with altered indigoidine production phenotypes^*a*^

Mutant identifier	Genes affected	Location	Direction	Pigment (% WT)	Growth rate (% WT)
Metabolism/growth					
57A	Citrate synthase, *gltA*	1,243	rev	26.4 ± 5.2	82.5 ± 2.4
10D	Malate dehydrogenase, *mdh*	936	fwd	34.4 ± 6.5	53.5 ± 2.4
53A	Cytochrome c oxidase I, *cco1*	929	rev	11.3 ± 2	96.7 ± 2.1
35A	Cytochrome c oxidase II, *cco2*	473	rev	35.9 ± 2.4	103 ± 2.6
45A	Cytochrome c oxidase II, *cco2*	538	fwd	26.7 ± 3.1	102 ± 3.4
65A	Cytochrome c oxidase I, *cco1*	−164	fwd	43.3 ± 6.4	93.5 ± 2.3
67B	Glucosamine-1-phosphate guanylyltransferase	−150	rev	18.3 ± 6	87.8 ± 3.3
61A	Ribose-phosphate pyrophosphokinase	−162	rev	44.2 ± 4.8	79.9 ± 4.1
Protein homeostasis					
101A	*dnaJ*	997	rev	1.2 ± 0.06	64.1 ± 1.8
7B1	*dnaK*	964	rev	1.6 ± 0.29	55.2 ± 2.1
41B	*dnaK*	246	rev	1.5 ± 2.2	53.4 ± 2.8
48A	*dnaK*	1,420	rev	1.4 ± 0.3	ND
62A	*dnaK*	720	rev	1.6 ± 1.6	ND
77A	*dnaK*	964	rev	0.7 ± 0.94	ND
101B	*dnaK*	165	rev	1.5 ± 0.23	60.5 ± 2.2
47A	*groES*	-3	rev	15.2 ± 1.3	90.0 ± 1.5
2A	*grpE*	77	rev	1.4 ± 1.5	56.7 ± 2.5
34A	*grpE*	154	rev	1.3 ± 0.16	48.4 ± 3.9
62B	*grpE*	170	rev	8.2 ± 0.5	ND
78B	*grpE*	−30	rev	54.3 ± 3	ND
36A	*clpA*	521	rev	**248 ± 14**	97.9 ± 2.3
79B	*clpA*	1,103	rev	**197 ± 13.8**	98.1 ± 1.7
6A	*clpP*	249	rev	**130 ± 2.4**	101 ± 2.8
39A	*clpP*	588	rev	**260 ± 18**	102 ± 3.4
39B	*clpP*	240	rev	**245 ± 6.3**	101 ± 3.6
Translation					
7B2	RNA helicase, *rhlE*	385	fwd	**218 ± 3.2**	94.3 ± 3.6
8B1	23S rRNA	255	rev	**121 ± 2.3**	96.1 ± 1.7
27A	23S rRNA	1,414	rev	**137 ± 2.8**	95.2 ± 1.3
32B	tRNA-Leu	−10	rev	**236 ± 3.3**	91.1 ± 2.4
76A	50S ribosomal protein L33	52	rev	35.7 ± 2.3	74.5 ± 4.4
33A	Ribosomal protein L9p	461	rev	84.4 ± 4.4	76.4 ± 4.1
Signaling/regulation					
8B2	*phoU*	474	rev	3.1 ± 0.8	79.8 ± 2.4
101C	ACSVIT_00025, *tciK*	240	rev	0.4 ± 0.6	95.8 ± 3.2
68B	*rpoN*	1,064	rev	39.5 ± 6.4	92.4 ± 3.1

^*a*^ Mutants are grouped into four functional categories. The location and direction of the transposon insertion are listed in base pairs relative to the start of the open reading frame, and relative orientation of the affected gene. Pigment production was measured on agar plate cultures and is listed as a percentage of the WT control (average ± standard deviation, *n* = 3 biological replicates). Values greater than 100% are bolded. Growth rate was measured for planktonic cultures in a 96-well dish assay and is listed as a percentage of the WT control (average ± standard deviation, *n* = 3 biological replicates).

Mutants fell into four broad groups based on the annotations of the genes affected. First, insertions impacting genes required for growth and/or metabolism showed reduced indigoidine production (Table 1). This is unsurprising given that producing a secondary metabolite requires energy and raw materials, in this case, glutamine. Among these were mutations in citric acid cycle genes, citrate synthase (*gltA*) and malate dehydrogenase (*mdh*), which we hypothesize limit the supply of amino acid precursors and/or energy for biosynthesis. A second group of mutants affected protein homeostasis pathways. This group was intriguing because mutations in chaperone genes (dnaJ, dnaK, and grpE) all eliminated pigment production, while those in bacterial proteasome components (*clpP* and *clpA*) increased indigoidine (Fig. 3A). A third group of mutants predicted to impact translation by affecting ribosomal RNA or biosynthesis genes, also showed significantly altered pigment production (Table 1). To limit the impact of growth rate differences on pigment quantification, the cell density of the inoculum was normalized in these experiments. Additionally, we measured the planktonic growth rate of the transposon mutants and compared it to WT (Table 1). These data showed reductions in growth rate for some mutants in metabolism and protein homeostasis. They indicate that there is not a simple relationship between planktonic growth rate and the level of pigment produced on agar, suggesting that reduced growth alone does not explain the phenotypes we observed. For example, four mutants predicted to affect translation had reduced growth rates but increased indigoidine levels (Table 1).

The final group of mutants impacted genes predicted to affect signaling and regulation. A mutation in *phoU* significantly reduced pigment production (Fig. 3A; Table 1). PhoU is a regulatory protein that modulates sensor histidine kinase activity in a range of bacteria, with potential roles in phosphate starvation and other stresses (25). A mutation in the alternative sigma factor *rpoN* also reduced pigment production. RpoN is known to regulate a wide range of genes in bacteria, including those involved in flagellar motility and nitrogen assimilation (26). Notably, RpoN is required for glutamine synthase expression in *Escherichia coli* (27), so a reduced supply of glutamine in this mutant could explain lower levels of indigoidine. The final gene of interest encodes a predicted sensor histidine kinase and is analyzed further below. The *igi* genes were conspicuously absent from the sequenced mutants. We know *igiD* is required for indigoidine production (Fig. 2B), and previous research implicated *igiB* and *igiC* as well (8). This suggests that additional transposon mutants need to be screened to fully saturate the genome.

### Complementation of the rpoN mutant

To validate one of the transposon mutants with a potential role in regulation, we cloned the rpoN gene into a broad host-range plasmid based on pCM62 (28) and transferred it into the mutant. The resulting strain had significantly increased indigoidine production relative to the mutant with an empty vector, but slightly less than the WT control (Fig. 3B). Additionally, we analyzed the swimming motility phenotype of these strains and found the *rpoN* mutant to have significantly reduced motility relative to the WT, which was restored in the complemented mutant (Fig. 3C). Collectively, these data support a role for rpoN in the positive regulation of indigoidine production and motility in *V. indigofera*.

### Two-component system regulates indigoidine production

**Fig 4:**
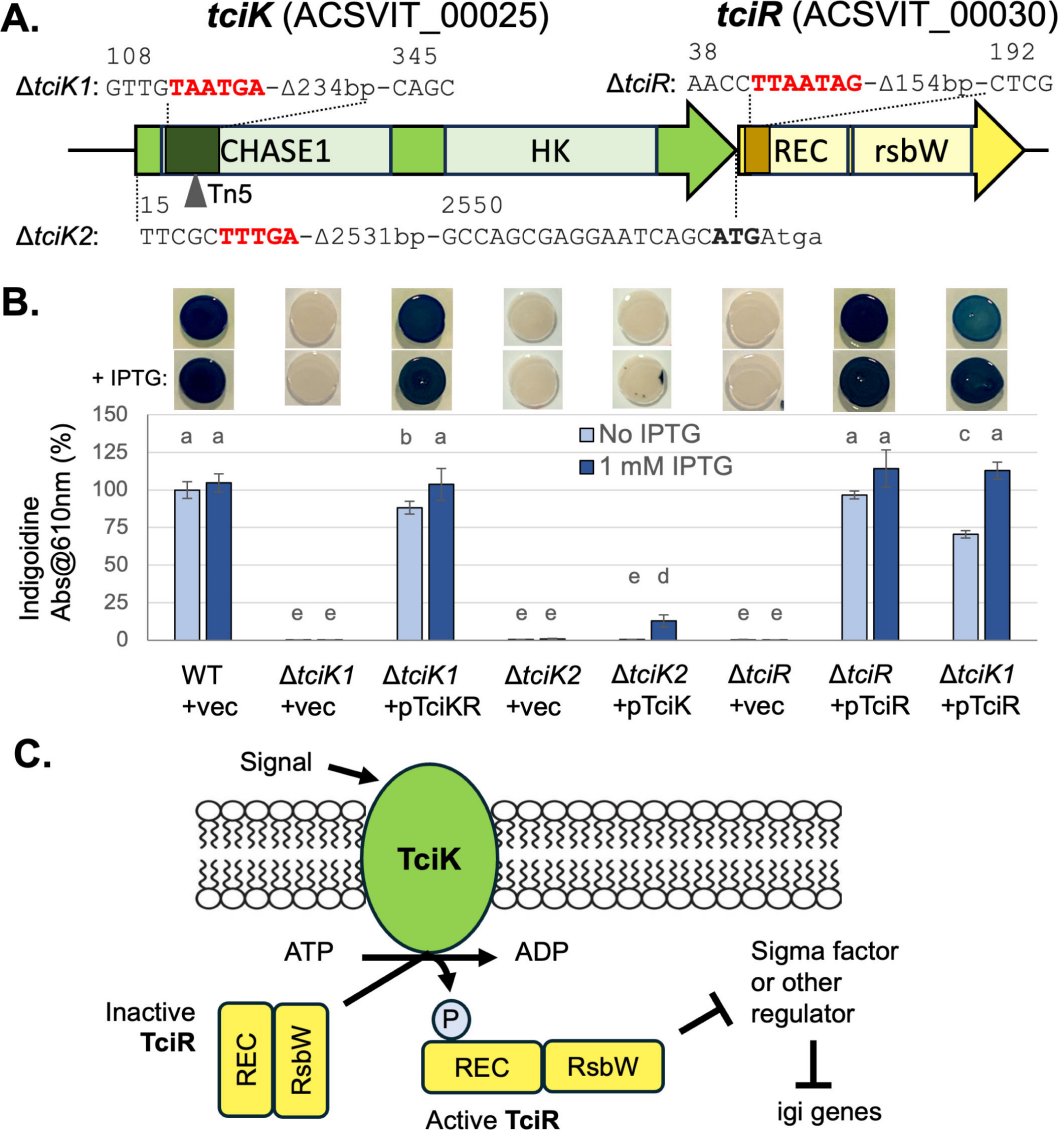
Non-canonical two-component system TciKR regulates indigoidine production. (**A**) The *tciKR* locus is shown at approximate scale with predicted protein domains on gene arrows. The transposon mutation in *tciK* is indicated by a triangle below, and genetic modifications to the locus are shown with numbers indicating the nucleotide position relative to the start of the reading frame and stop codons added to the reading frame shown in red. In the diagram for the Δ*tciK2* mutant, the predicted start codon for *tciR* appears in bold and the stop codon for *tciK* is lowercase. (**B**) Indigoidine production under conditions with and without IPTG, shown as a percent of WT in the No IPTG condition (mean ± standard deviation), was quantified by spectrophotometry. Strains labeled + vec contain pSRKKm, while other plasmids contain the indicated gene under IPTG-inducible control. Different lowercase letters above the bars indicate *P* < 0.05 in pairwise *t*-tests after Bonferroni correction; *n* = 6 from three independent experiments. Representative images of spot cultures growing on TSA plates with or without IPTG are shown above. (**C**) A model for how the TciKR system regulates indigoidine production. TciK senses an environmental signal and, in response, phosphorylates TciR’s REC domain. Phosphorylated TciR is active to regulate cellular outputs via its RsbW-like domain, which may include derepression of igi gene expression via inhibition of a sigma factor or other regulator.

A transposon insertion in ACSVIT_00025 completely abolished indigoidine production (Table 1). This gene encodes a predicted sensor histidine kinase and forms an apparent two-gene operon with a predicted response regulator ACSVIT_00030 (Fig. 4A). To investigate their functions, we constructed deletion strains for each gene and found that disrupting either one eliminated pigmentation (Fig. 4B). Based on these data, we named the genes *tciKR* for two-component regulator of indigoidine, kinase, and regulator. We attempted to complement the Δ*tciK1* mutant with *tciK* on a plasmid under IPTG-inducible control but found this did not restore any pigment production (data not shown). This led us to hypothesize that the mutation may have polar effects on *tciR*. To test this, we expressed the *tciKR* operon from the same plasmid and found it restored indigoidine production to Δ*tciK1,* with full complementation observed with the addition of IPTG (Fig. 4B). This result supported a role for *tciR* in pigment production but left open the possibility that mutations in *tciK* only impact the phenotype through effects on the downstream gene and not via the function of the TciK protein. Mutating *tciR* alone eliminated pigment production, and this mutant was fully complemented with the introduction of *tciR* on a plasmid (Fig. 4B). Sequencing the *tciR* gene in the Δ*tciK1* background found no mutations in the *tciR* reading frame, suggesting the Δ*tciK1* mutation impacts *tciR* expression (data not shown).

We built another mutant, Δ*tciK2*, to see if a more complete removal of the *tciK* reading frame would allow for *tciR* expression and clarify if TciK plays a role in pigment regulation (Fig. 4A). Like the first mutant, Δ*tciK2* did not produce pigment. However, this strain was partially complemented by the reintroduction of *tciK* on a plasmid (Fig. 4B). Pigment production by Δ*tciK2* + p*tciK* was about 12.5% of WT and only observed when the inducer was added. Strikingly, the pigment was not uniformly distributed in colonies but instead occurred in puncta (Fig. 4B). This result was observed consistently, even after cultures were propagated from the blue or white sectors of spot cultures (Fig. S1), suggesting that the non-uniform pigment phenotype is stable and not the result of spontaneous mutations. Altogether, genetic analyses of *tciK* and *tciR* indicate that these genes regulate indigoidine production.

Searching the Conserved Domain Database (29) with the TciR protein sequence revealed it to be unusual because in place of a typical C-terminal DNA-binding domain, it has a predicted anti-sigma factor domain with similarity to RsbW, a regulator of the SigB-dependent stress response in Gram-positive bacteria (30). The Conserved Domain Architecture Retrieval Tool (31) retrieved 100 proteins with this domain architecture: 74 from Proteobacteria, 13 from Planctomycetes, 5 from Spirochetes, and the remainder from a range of other groups. This suggests a broad distribution of TciR-like proteins in bacteria. To further investigate TciR’s conservation and distribution, we performed a blastP search of the refseq database. This returned 272 proteins with ≥90% query coverage and ≥35% AAI. These putative homologs of TciR are found in a wide range of beta-proteobacterial species, but also many gamma- and alphaproteobacteria. More distantly related taxa with TciR-like proteins include Nitrospira, Desulfonema, Magnetofaba, and several *Leptospira* species.

A basic model for how this two-component system may regulate indigoidine production is that TciK senses an extracellular signal and phosphorylates TciR in response (Fig. 4C). Phospho-TciR could then inhibit a sigma factor via its RsbW-like domain, altering gene expression. Given that most sigma factors act as transcriptional activators, regulation of indigoidine production at the level of *igiD* transcription by this system is probably indirect. Alternative models are discussed below. To test whether TciR acts downstream of TciK in this signaling pathway, we overexpressed *tciR* in the Δ*tciK1* strain. We reasoned that if TciR acts downstream of TciK, overexpression of the regulator may compensate for the absence of the kinase. Consistent with this prediction, we found that indigoidine levels were partially restored in Δ*tciK1* with the addition of pTciR in the absence of inducer and fully restored when IPTG was added (Fig. 4B). These results support the hypothesis that TciR acts downstream of TciK.

## DISCUSSION

In this preliminary characterization of *V. indigofera* OSW_575, we sought to develop an experimental system for investigating how and why bacteria produce indigoidine. This effort succeeded, as we found the strain to be amenable to genetic manipulation and we were able to uncover a range of genes that impact pigment production. Among them was a two-component system consisting of a sensor histidine kinase and a response regulator with a non-canonical domain architecture. Here we discuss our interpretations of the genetic screen results, as well as future directions for investigating this system.

*V. indigofera* OSW_575 was isolated from the terminus of a stormwater drainage pipe. This fits with prior reports finding *Vogesella* in freshwater environments subject to anthropogenic disturbance (5). Phylogenetic analysis and genome-wide sequence comparisons showed our strain to be closely related to *V. indigofera* representatives with available genome sequences, as well as to two strains that have not yet been assigned a species name: *Vogesella* sp. AC12 and *Vogesella* sp. EB (Fig. 1). These strains, and most of the *V. indigofera* isolates included in our analysis, were isolated from surface freshwater across three different continents, suggesting a cosmopolitan distribution for this species. We found that pairwise ANI comparisons of *V. indigofera* strains gave values >95.4%, consistent with them being the same species (see reference 18; Table S1), but some dDDH comparisons gave values that fell below the species threshold. The reason for this discrepancy is unclear. Notably, the dDDH values cluster *V. indigofera* strains into two distinct groups that are mirrored in the multi-locus phylogeny based on core protein sequences (Fig. 1). The significance of this split deserves investigation. Further characterization of these and other strains of *V. indigofera* may reveal that reclassification of some of them is warranted. A comparative genomic study of the cosmopolitan freshwater betaproteobacterium *Polynucleobacter asymbiotica* showed a high degree of genomic cohesion (ANI > 97%) among strains from geographically distant sites (32), but the authors did not include dDDH analysis. Limited conclusions can be drawn from our small sample size, but it is notable that some *V. indigofera* isolates from the same river in Southwest China (e.g., LYT24W and LYT23W) fall into separate clades and have dDDH ~60%. This suggests that if speciation is occurring between them, it is not due to geographic isolation.

Comparing the gene content of *V. indigofera* OSW_575 to that of its closest sequenced relatives, we found that the capacity for indigoidine production is not universal for this species (Fig. 1), nor does it correlate with phylogenetic clades discussed above. Instead, key genes in the biosynthetic locus (*igiA-D*) are absent from genomes of four out of ten *V. indigofera* strains. This is consistent with the lack of pigmentation in these strains, reported by Lu et al., who isolated and characterized a majority of *V. indigofera* strains with publicly available genome sequences (21). Comparing the *igi* loci of sister strains that are discordant for pigment production reveals likely deletions of *igi* genes from non-pigmented strains (Fig. S2). Given that the functions of indigoidine in *V. indigofera* remain ill-defined, the significance of its maintenance/loss in different strains is an exciting area for future research.

Our screen for transposon mutants with altered pigment production identified many that impact protein homeostasis pathways (Table 1). Interestingly, chaperone genes are required for pigment production, while the bacterial proteasome (*clpAP*) appears to inhibit it. These phenotypes could be due to a direct role for protein homeostasis systems in modulating the stability of IgiD or other proteins needed for indigoidine biosynthesis. Alternatively, perturbing protein homeostasis could impact cellular regulation, in part because these systems control the degradation of transcriptional regulator proteins, including sigma factors (30, 33). The apparent requirement of DnaJ, DnaK, and GrpE for indigoidine production is notable because these proteins work together in *E. coli* to counter misfolded proteins during heat stress (33). They also regulate the activity of the sigma factor RpoH by sequestering it under normal conditions, then releasing it in response to increasing numbers of misfolded proteins (33). Based on the phenotypes of these mutants in *V. indigofera* and what is known from *E. coli*, we hypothesize that RpoH inhibits indigoidine expression and that loss of the chaperones leads to constitutive RpoH activity. Mutations in *clpA* and *clpP* are also expected to impact the proteome and global regulation. These changes may include the accumulation of a positive regulator of indigoidine synthesis, given the hyperpigmentation we see in these mutants (Fig. 3A). These hypotheses will be tested in future studies. In support of a regulatory role for *clpA*, Cude et al. found *clpA* transposon mutants of *Phaeobacter* show hyperpigmentation and increased *igiD* transcription (8); they also observed some pigment production in liquid cultures of *clpA*, which was absent from the WT strain. We also observed slight pigmentation in *clpP* and *clpA* mutant liquid cultures (Fig. S3). However, the phenotype defied quantification as it was subtle and inconsistent. None of the other mutant strains with elevated pigment levels on plates exhibited pigment production in liquid cultures. This suggests a robust regulation mechanism limits pigment production in liquid culture, which will be a focus for future work.

Some of the transposon mutants that we recovered only once, for example, *phoU*, represent interesting leads that require further validation. We note that caution should be used when drawing conclusions from the phenotypes of single transposon mutants because additional, unmapped mutations could also be present. We expect that further transposon mutant screening will validate some of these by uncovering additional hits and may also reveal more genes of interest, given that we did not fully saturate the genome.

Genetic analysis of the *tciKR* locus supports a model in which the TciK sensor histidine kinase acts upstream of the TciR response regulator to regulate indigoidine production (Fig. 4C). We present data from two mutants with different portions of the *tciK* reading frame deleted. One could not be complemented by the addition of *tciK* in *trans*, and the other was only partially complemented. The reason for this is unclear, as is the basis for the non-uniform pigment production in Δ*tciK2* + pTciK (Fig. 4B). The fact that indigoidine production in this background is dependent on IPTG induction of *tciK* provides strong evidence for *tciK’s* role in the pathway. We hypothesize that *tciR* expression is impacted in both *tciK* deletion strains. The location of promoters in this region is unknown, and it is possible that the regulation of these genes is complex. More work is needed to investigate if *tciK* and *tciR* form an operon and/or whether their transcriptional regulation is coordinated.

Homologs of TciR are widespread in bacteria, including many that do not make indigoidine, suggesting it has a broadly conserved regulatory function. TciR is atypical in that it combines an N-terminal receiver domain and a C-terminal, RsbW-like anti-sigma factor domain. To our knowledge, no protein with this architecture has been functionally characterized, though they were noted in a census of response regulator variants (34). In *Bacillus subtilis* and some other Gram-positive bacteria, RsbW (which lacks a REC domain) binds to and inhibits the sigma factor Sigma B, blocking the expression of stress response genes (30). Induction of the Sigma B regulon is triggered through a partner-switching mechanism in which the STAS-domain protein RsbV binds to RsbW, freeing up Sigma B to associate with RNA polymerase. Partner switching is regulated by the phosphorylation state of RsbV, which is inactivated by RsbW’s kinase activity and activated by several phosphatases (35). While the amino acids required for kinase activity appear to be conserved in TciR, this exact mechanism is unlikely to function in *V. indigofera* because it lacks a homolog of RsbV.

Alternatively, TciR’s RsbW-like domain may target a protein or proteins other than a sigma factor. This is the case for regulatory systems found in other Gram-negative bacteria that include RsbW-like proteins (36). For example, the SypE protein of *Vibrio fischeri* includes an RsbW-like kinase domain, a REC domain, and a PP2C phosphatase domain (37). SypE’s opposing kinase and phosphatase activities control the phosphorylation state of STAS-domain protein SypA, which in turn promotes biofilm formation through an unknown post-transcriptional mechanism (38). Future work will investigate the structure and function of TciK and TciR with the goal of understanding regulatory mechanisms conserved in TciR-like proteins. This will include establishing whether phosphorylation of TciR’s REC domain by TciK controls its activity, whether the RsbW-like domain can act as a kinase, and identifying potential substrates and/or binding partners of TciR. Finally, we aim to determine how the TciKR system impacts indigoidine production and whether it occurs at the transcriptional or post-transcriptional level.

In conclusion, despite being known to science for well over a century, very few studies have focused on *V. indigofera*. This study establishes strain OSW_575 as a useful experimental system for investigating the production and regulation of indigoidine. We showed that pigment production by this strain requires the indigoidine synthase gene *igiD*, and that transposon mutations impacting protein homeostasis and other cellular processes alter indigoidine levels. We performed a genetic analysis of the novel two-component system composed of sensor histidine kinase *tciK* and a non-cannonical response regulator *tciR*, showing that these genes regulate pigment production. TciR-like proteins are conserved across a diverse group of organisms, so further characterization of this system is likely to uncover new insights into regulatory mechanisms in bacteria.

## MATERIALS AND METHODS

### Strains and growth conditions

Strains and plasmids are listed in Table 2. *V. indigofera* OSW_575 was isolated from stormwater drain effluent (GPS coordinates: 43.456932°N 76.541966°W) on modified R2A plates (0.2 g L^−1^ peptone, 0.8 g L^−1^ casamino acids, 0.5 g L^−1^ glucose, 0.3 g L^−1^ dipotassium phosphate, 0.6 mM MgSO_4_, and 15 g L^−1^ agar) after a 48-h incubation at room temperature. Routine culture of this strain was performed at 28°C on modified tryptic soy agar (TSA) plates (20 g L^−1^ Tryptic Soy Broth Powder from Becton Dickinson, 15 g L^−1^ agar), or shaking at 250 rpm (same medium, without agar). *E. coli* was grown at 37°C in LB (10 g L^−1^ tryptone, 5 g L^−1^ yeast extract, and 5 g L^−1^ NaCl). Antibiotics were used when necessary: 50 µg mL ^−1^ kanamycin and 10 µg mL ^−1^ tetracycline. *S. cerevisiae* was grown at 30°C on YPD-lite (8 g L^−1^ yeast extract, 8 g L^−1^ peptone, 8 g L^−1^ dextrose, and 16 g L^−1^ agar) or complete supplemental mixture without uracil (20 g L^−1^ dextrose, 20 g L^−1^ agar, 6.7 g L^−1^ yeast nitrogen base, and 2 g L^−1^ CSM-Uracil). IPTG stock solution was made in deionized water and added at the final concentrations indicated. Swimming motility of *V. indigofera* was assayed in TSA with 3 g L^−1^ agar and quantified, as described previously (39). To generate images of indigoidine phenotypes, 10 µL aliquots of overnight culture was spotted onto TSA plates and incubated at room temperature for 24 h.

**TABLE 2:** Strains and plasmids used in this study

Strain	Relevant characteristics	Source
*Escherichia coli* S17-lpir	Conjugation donor	(40)
*Saccharomyces cerevisiae -URA*	Uracil auxotroph	(41)
*Vogesella indigofera* OSW_575	Wild type	This study
*V. indigofera* Δ*igiD*	Indigoidine synthase mutant	This study
*V. indigofera* Δ*ticK1*	*tciK* mutant with small deletion	This study
*V. indigofera* Δ*ticK2*	*tciK* mutant with large deletion	This study
*V. indigofera* Δ*ticR*	response regulator mutant	This study

Plasmid	Relevant characteristics	Source
pMQ460	Allelic exchange vector, Km^r^	(42)
pMQ460_igiDKO	*igiD* knockout construct, Km^r^	This study
pMQ460_tciKKO1	*tciK1* knockout construct, Km^r^	This study
pMQ460_tciKKO2	*tciK2* knockout construct, Km^r^	This study
pMQ460_tciRKO	*tciR* knockout construct, Km^r^	This study
pRL27	Tn5 delivery vector, Km^r^	(43)
pDN5	Tet^r^, AraC P^BAD^ SceI	(44)
pSRKKm	Shuttle plasmid, IPTG-inducible P^lac^, Km^r^	(23)
pIgiD	pSRKKm with IPTG-inducible *igiD*	This study
pTciKR	pSRKKm with IPTG-inducible *tciKR*	This study
pTciK	pSRKKm with IPTG-inducible *tciK*	This study
pTciR	pSRKKm with IPTG-inducible *tciR*	This study
pCM62	Shuttle plasmid, Tet^r^	(28)
pMQ87	Yeast origin CEN, and selection URA3	(41)
pCM62Y	pCM62 with added CEN and URA3	This study
pMQ71b	araC and P^BAD^	(45)
pOSW4	CEN, URA3, araC, P^BAD^, and Tet^r^	This study
pRpoN	pOSW4 with *rpoN*	This study

### Genome sequencing and analyses

Genomic DNA was isolated with the Qiagen DNeasy kit, following the manufacturer’s instructions, and 150 bp paired-end Illumina sequencing performed by SeqCenter (Pittsburgh, PA). The sequence was assembled using SPAdes, annotated using RASTtk, and analyzed with Quast, CheckM, BLAST, and FastANI, all implemented within KBase using default settings (46). The genome sequence of *V. indigofera* OSW_575 described in this study has been deposited at DDBJ/ENA/GenBank under the accession JBPXRZ000000000. Sequences for tciK and tciR have also been deposited in GenBank and given accessions PX409052 and PX409053, respectively.

### Comparative genomics and phylogenetics

RASTtk v1.073 (47) was used to annotate the *V. indigofera* OSW_575 genome, and those of 24 related bacteria (listed in Fig. 1). The genomes chosen for comparison included all *V. indigofera* genomes available in GenBank at the time we initiated our study (October 2023), representatives of nine other *Vogesella* species, and six more distantly related Chromobacteriaceae. For phylogenetic and gene content analyses, OrthoMCL was used to build a pangenome in KBase, determining clusters of orthologous proteins, and PhyloMarker was used to identify and extract sequences of single-copy marker proteins (48, 49). Amino acid alignments for each protein cluster were performed with mafft and trimmed with trimAL set to gappyout (50, 51). ProtTest3 was used to select the best model for each alignment using Bayesian Information Criteria (52). Finally, the trimmed alignments were used to build a phylogenetic tree in iqtree2 using a partitioned best-fit model for each of the alignments and 100 bootstraps (53). The tree was visualized using iTOL (54).

### Gene deletion via allelic replacement

Mutations in *igiD*, *tciK*, and *tciR* were built using the allelic replacement plasmid pMQ460, and Sce-I expressing plasmid pDN5 as previously described (44). Specific sequence alterations are diagrammed in the corresponding figures. Briefly, ~1 kb DNA fragments from upstream and downstream of the targeted gene were amplified by PCR and cloned adjacent to each other in pMQ460. Completed allelic exchange constructs were introduced into *V. indigofera* via conjugation with *E. coli* S17 λ-pir, and single-crossover integrants were selected for on modified R2A containing 50 µg mL ^−1^ kanamycin. After re-streaking on selective plates, integrants received the pDN5 plasmid via conjugation, and double-crossover mutants were selected for on modified R2A containing 10 µg mL ^−1^ tetracycline, 0.2% arabinose, and sucrose taking the place of glucose in the recipe. Deletion mutations were confirmed by PCR and sequencing.

### Complementation plasmids

Genes were cloned into pSRKKm via PCR, restriction digest, and ligation. Primers utilized in this study are listed in Table S4 along with the restriction enzyme sites that were used. The *rpoN* complementation plasmid was built as follows. The yeast origin CEN and selectable marker URA3 were amplified as one PCR product from pMQ87 using primers that introduced 25–30 bp homologous to destination plasmid pCM62. This broad-spectrum bacterial shuttle vector was linearized with AfeI and, along with the CEN-URA3 product, was used to transform *S. cerevisiae*, as described (41). The resulting plasmid (pCM62Y) was linearized with KpnI, for the addition of an AraC P^BAD^ cassette, which was amplified from pMQ71b. Upon transformation of yeast and recombination, this yielded pOSW4. pOSW4 was linearized with SphI, and *rpoN* was cloned into the junction via recombination in yeast. Candidate plasmids were confirmed by restriction digest and sequencing, then introduced into *V. indigofera* by conjugation.

### Indigoidine measurement

We used a semi-quantitative assay for measuring pigment produced by bacteria grown on filter paper. Whatman filter paper discs 2.5 cm in diameter were cut into quarters and sterilized by autoclaving, then applied to the surface of TSA plates. Bacteria were grown in TSB overnight, then cell density was measured as OD 600 nm and normalized to the WT strain to equalize each inoculum. 15 µL of the cell suspension was added directly to the paper, and plates were incubated at 24.5°C for 24 h. To recover and solubilize pigment, each filter was placed in a 2 mL screw-capped tube with 100 µL of 0.5 mm glass beads and 750 µL of 9:1 dimethyl sulfoxide:acetone solvent. Tubes were homogenized on a bead mill for two 1 min intervals with 1 min rest in between. Debris was pelleted by centrifugation at 16,000 × *g* for 5 min, and supernatant was withdrawn for analysis. Absorbance at 610 nm was measured for three 100 µL aliquots per filter in a 96-well dish. After subtraction of the blank, triplicate absorbance values were averaged to obtain a single observation.

### Transposon mutagenesis and mapping

Transposon mutants of *V. indigofera* were generated via conjugal transfer of the mini-Tn5 vector pRL27 from *E. coli*, as described (55). Briefly, cells from overnight cultures of donor and recipient were washed twice in fresh TSB medium before mixing and incubation on TSA at 28°C for 4 h. Cells were collected and diluted 10-fold in phosphate-buffered saline before plating on R2A agar with 50 µg mL ^−1^ kanamycin to select for mutants and minimize the growth of the donor. Colonies were visually screened after 5 days, then isolated for repeated testing. Genomic DNA was isolated from mutants of interest using the Promega Wizard DNA isolation kit as per the manufacturer’s instructions. Transposon insertion sites were mapped by sequencing of arbitrarily-primed PCR products, as described (56). PCR was performed in two rounds with Onetaq polymerase, first with primers Arb1, Arb6, and Extsx, and second with Arb2 and Intsx. Products were purified with the Thermo GeneJet PCR purification kit and sequenced with primer Intsx. Transposon junctions were identified, and adjacent sequences were matched to the genome via blastn.

### Growth rate measurement

Planktonic growth rate was measured in a 96-well assay adapted from Kurokawa and Ying (57). Cultures were grown in 3 mL of TSB in test tubes for 20 h before being subcultured at a 1:20 dilution in 100 µL of fresh TSB in a 96-well plate. Triplicate technical replicates of each strain were included in each of three biological replicates, which were performed on different days, and the position of the samples in the plates was randomized to avoid positional bias. The plate was incubated at 28°C, shaken on the low setting, and absorbance read at 600 nm every 30 min for 16 h using a Biotek Powerwave XS microplate reader. The growth rate was calculated on a per-well basis as an average across the three hours where the rate of absorbance increase was greatest. Technical replicate rates were averaged, and then the average and standard deviation of biological replicates are reported in Table 1.

## Data Availability

The genome sequence of *V. indigofera* OSW_575 described in this study has been deposited at DDBJ/ENA/GenBank under the accession JBPXRZ000000000. Sequences for tciK and tciR have also been deposited in GenBank and given accessions PX409052 and PX409053, respectively.

## SUPPLEMENTAL MATERIAL

Supplemental tables and figures jb.00364-25-s0001.pdf Tables S1 to S4 and Figures S1 to S3.

ASM does not own the copyrights to Supplemental Material that may be linked to, or accessed through, an article. The authors have granted ASM a non-exclusive, world-wide license to publish the Supplemental Material files. Please contact the corresponding author directly for reuse.
