# Supplementary material for: Regulation of indigoidine production in Vogesella indigofera by a novel two-component system
Source: J Bacteriol. 2025 Dec 3;208(1):e00364-25. doi: 10.1128/jb.00364-25 (PMC12826045; doi:10.1128/jb.00364-25)
Supplement: Supplemental tables and figures — Tables S1 to S4 and Figures S1 to S3. [file jb.00364-25-s0001.pdf]

Supplementary File for "*Regulation of Indigoidine Production in* Vogesella indigofera *by a Novel Two-component System*" by Babcock et al.

Table S1. Calculated average nucleotide identity. Average nucleotide identity (ANI) was calculated with fastANI using genome sequences from our strain (Osw_575), seven strains of *V. indigofera* (listed by strain designation) and three other closely related bacteria: *Vogesella sp.* AC12, *Vogesella sp.* EB, and *Vogesella fluminis* KCTC 23713.

	**Osw_ 575**	**DSM 3303**	**LYT 10W**	**LYT 16W**	**LYT 22W**	**LYT 23W**	**LYT 24W**	**SH 7W**	**V_ AC12**	**V_ EB**	**V_ flu**
**Osw_575**	100.0	95.46	96.76	96.19	96.98	95.95	95.52	95.53	95.56	95.55	93.46
**DSM_3303**	95.42	100.0	95.43	95.28	95.41	95.19	97.25	97.22	97.30	97.30	93.15
**LYT10W**	96.71	95.44	100.0	96.22	96.91	96.09	95.38	95.38	95.37	95.52	93.50
**LYT16W**	96.16	95.26	96.28	100.0	96.34	96.72	95.32	95.36	95.28	95.32	93.30
**LYT22W**	96.98	95.50	96.90	96.37	100.0	96.08	95.53	95.58	95.48	95.44	93.42
**LYT23W**	95.94	95.16	96.11	96.77	96.09	100.0	95.26	95.31	95.22	95.25	93.17
**LYT24W**	95.51	97.24	95.41	95.33	95.51	95.31	100.0	97.13	97.33	97.29	93.18
**SH7W**	95.49	97.14	95.39	95.35	95.49	95.26	97.12	100.0	97.23	97.17	93.09
**V_AC12**	95.52	97.29	95.39	95.16	95.43	95.17	97.29	97.17	100.0	97.28	93.04
**V_EB**	95.47	97.28	95.44	95.37	95.51	95.23	97.27	97.23	97.33	100.0	93.11
**V_flu**	93.50	93.19	93.43	93.24	93.38	93.31	93.07	93.18	93.14	93.09	100.0

Table S2. Calculated average amino acid identity. Average amino acid identity (AAI) was calculated using EzAAI. Genomes compared are the same as in Table S1.

	**Osw_ 575**	**DSM 3303**	**LYT 10W**	**LYT 16W**	**LYT 22W**	**LYT 23W**	**LYT 24W**	**SH 7W**	**V_ AC12**	**V_ EB**	**V_ flu**
**Osw_575**	100.0	97.11	97.96	97.40	98.24	97.35	97.01	97.15	97.03	97.25	94.81
**DSM_3303**	97.11	100.0	97.13	96.99	97.20	96.90	98.36	98.36	98.33	98.48	94.39
**LYT10W**	97.96	97.13	100.0	97.51	98.03	97.50	97.06	97.12	97.15	97.19	94.72
**LYT16W**	97.40	96.99	97.51	100.0	97.63	97.89	96.90	96.91	96.94	97.04	94.56
**LYT22W**	98.24	97.20	98.03	97.63	100.0	97.63	97.19	97.13	97.21	97.32	94.71
**LYT23W**	97.35	96.90	97.50	97.89	97.63	100. 0	96.93	97.00	96.87	97.04	94.50
**LYT24W**	97.01	98.36	97.06	96.90	97.19	96.93	100. 0	98.23	98.42	98.43	94.42
**SH7W**	97.15	98.36	97.12	96.91	97.13	97.00	98.23	100. 0	98.34	98.46	94.35
**V_AC12**	97.03	98.33	97.15	96.94	97.21	96.87	98.42	98.34	100.0	98.48	94.32
**V_EB**	97.25	98.48	97.19	97.04	97.32	97.04	98.43	98.46	98.48	100.0	94.52
**V_flu**	94.81	94.39	94.72	94.56	94.71	94.50	94.42	94.35	94.32	94.52	100.0

Table S3. Calculated digital DNA-DNA hybridization. Digital DNA-DNA hybridization was calculated using the TYGS server. Genomes compared are the same as in Table S1.

	**Osw_ 575**	**DSM 3303**	**LYT 10W**	**LYT 16W**	**LYT 22W**	**LYT 23W**	**LYT 24W**	**SH 7W**	**V_ AC12**	**V_ EB**	**V_ flu**
**Osw_575**	100	60.9	70.5	66.6	72.6	65.2	60.8	61.2	61.3	61	50.6
**DSM_3303**	60.9	100	60.8	60.6	61.1	59.8	74.9	74.7	75.9	75.9	49.2
**LYT10W**	70.5	60.8	100	67.2	71.7	66.1	60.6	60.8	60.7	61.2	50.3
**LYT16W**	66.6	60.6	67.2	100	67.3	71.3	60.1	60	60.2	60.8	49.7
**LYT22W**	72.6	61.1	71.7	67.3	100	66.2	60.8	61.1	60.8	61.2	50.5
**LYT23W**	65.2	59.8	66.1	71.3	66.2	100	59.9	60.1	59.8	60	49.3
**LYT24W**	60.8	74.9	60.6	60.1	60.8	59.9	100	74.3	75.4	75.3	49.1
**SH7W**	61.2	74.7	60.8	60	61.1	60.1	74.3	100	75	75.3	49.1
**V_AC12**	61.3	75.9	60.7	60.2	60.8	59.8	75.4	75	100	75.8	48.9
**V_EB**	61	75.9	61.2	60.8	61.2	60	75.3	75.3	75.8	100	49.2
**V_flu**	50.6	49.2	50.3	49.7	50.5	49.3	49.1	49.1	48.9	49.2	100

Figure S1. Non-uniform pigment production in spot cultures of Δ*tciK2*+p*tciK*. We sought to test if the non-uniform production of pigment in spot cultures of this strain was a stable phenotype and not the result of spontaneous mutations. Pigmented and non-pigmented sectors of two successive spot cultures were streaked to isolation, then liquid cultures were grown from single colonies and spotted onto new plates. Each time, spot cultures exhibited the non-uniform pigment phenotype regardless of whether the source was white or blue growth. Pigment production was always dependent on IPTG induction of *tciK*.

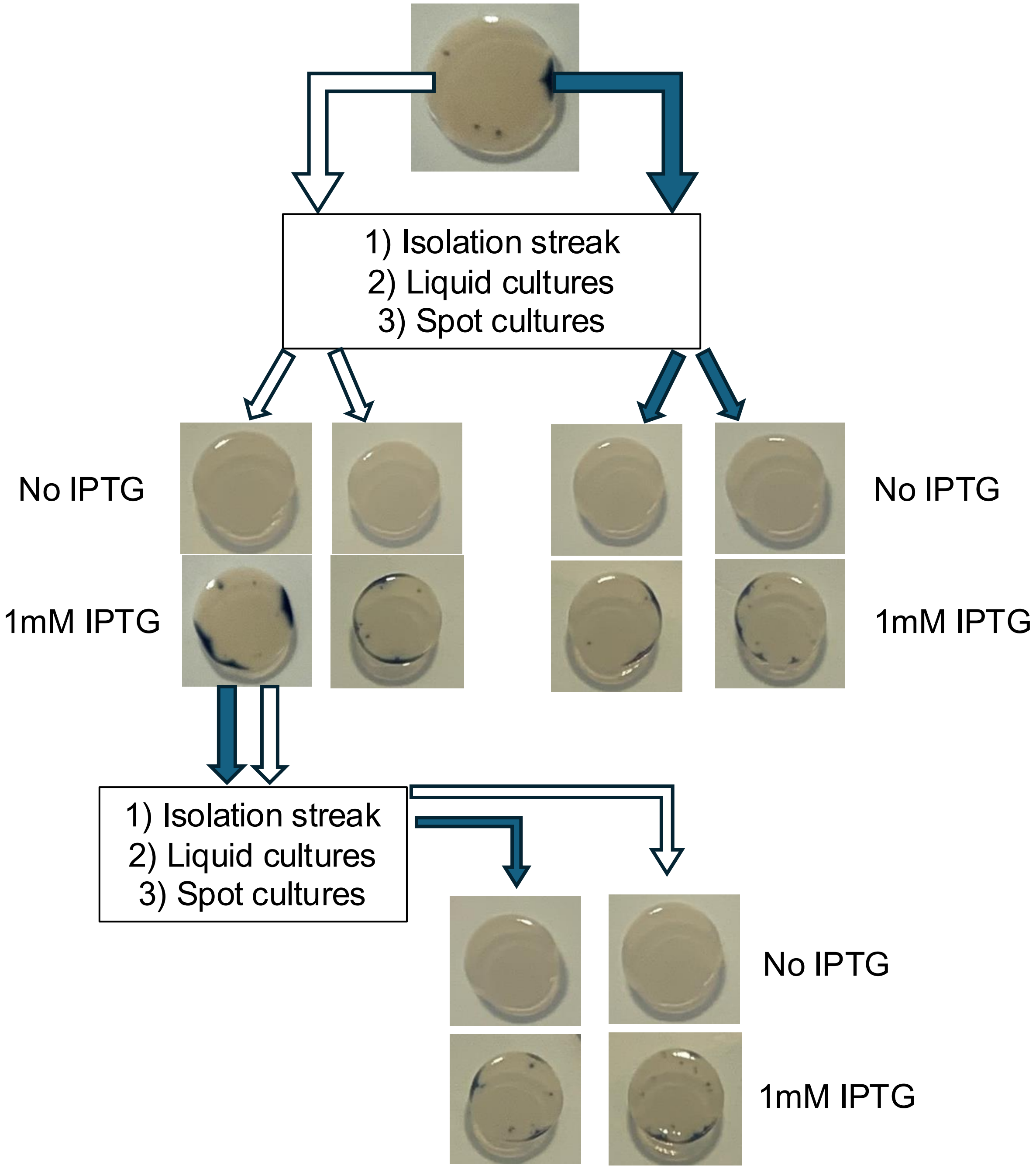

Figure S2. Loss of indigoidine synthesis (*igi*) genes from *V. indigofera* strains. Gene contents of two pairs of closely related strains are compared. The strain designations are labeled on the left in boxes. Grey boxes with red outline signify that the strain is missing some igi genes and does not produce pigment. Blue boxes signify that the strain has a full complement of igi genes and produces pigment. The number underneath each label is the NCBI accession number for the contig analyzed. Gene prediction and visualization was performed in Kbase, and the nucleotide positions in the contig are numbered above. Diagonal lines connect similar sequences to illustrate potential gene loss from the grey strains relative to the blue ones. Single-letter gene labels are A=*igiA*, B=*igiB*, C=*igiC*, R=AcrR family Transcriptional regulator, F=Flavodoxin reductase, and L=Histidine ammonia-lyase.

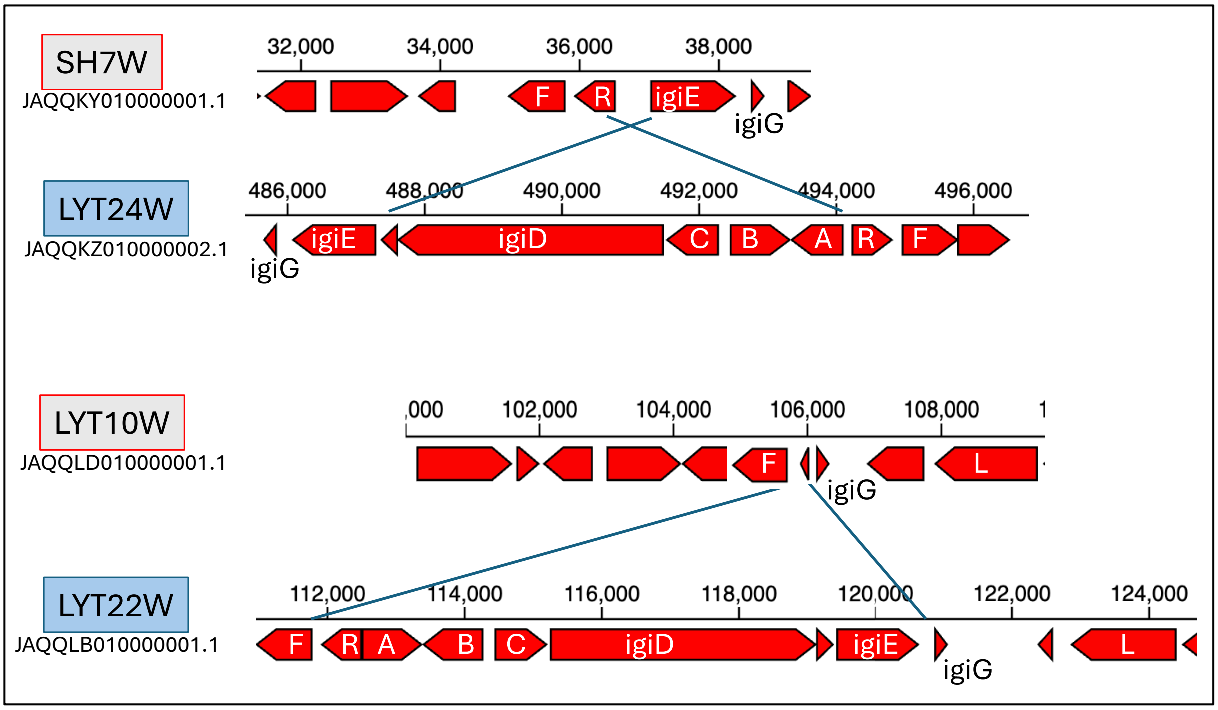

Figure S3. Pigment production in liquid cultures by *clpA* and *clpP* mutants. Overnight cultures of transposon mutants *clpA*_36A (middle) and *clpP*_39B (right) are shown next to a wildtype control carrying the pSRKKm plasmid (left). All the strains were grown at 28°C in TSB with 50 μg ml^{-1} kanamycin for 20 hours, shaking at 250 rpm. *Note:* production of pigment in liquid was inconsistent and only observed when cultures were started from fresh isolation streaks (24-48 hrs. old).

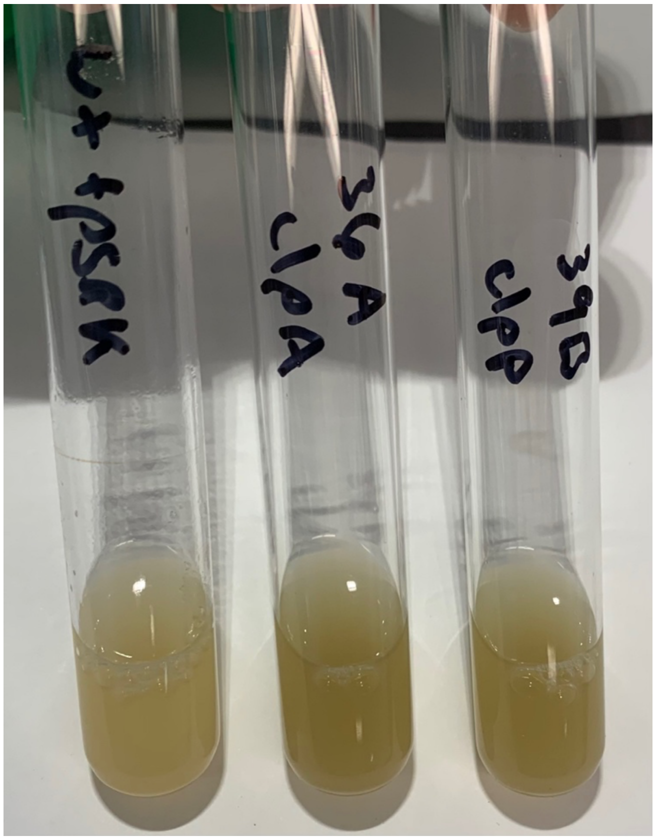

Table S4. Oligonucleotide primers used in this study. Annealing bases are capitalized. Bases used in recombination cloning are lowercase. Restriction sites used in the study are bolded.

**Name**	**Sequence**
Extsx	GACAACAAGCCAGGGATG
Intsx	CGCACTGAGAAGCCCTTAGAGC
Arb1	GGCCACGCGTCGACTAGTACN$_{10}$GATAT
Arb2	GGCCACGCGTCGACTAGTAC
Arb6	GGCCACGCGTCGACTAGTACN$_{10}$ACGCC
igiDKO up fwd	caagcttgcatgcctgcaggtcgactctagaggatcGGTCCGCGATTTCCATCGCTTG
igiDKO up rev	agcaccagcttgacctcattaACGCAGATACGGACTGGTGTGG
igiDKO dwn fwd	agtccgtatctgcgttaatgaGGTCAAGCTGGTGCTCACCAAC
igiDKO dwn rev	cagctatgaccatgattacgaattcgagctcggtaccCGTCCAGCTCGATACGGTAGCC
tciKKO up fwd	caagcttgcatgcctgcaggtcgactctagaggatcCCAGCATGCGTCGCGTTGTATTC
tciKKO up rev	gatcaccgtctgctgtcattaCAACTGCTGCTGCTCGCTGTC
tciKKO dwn fwd	gagcagcagcagttgtaatgaCAGCAGACGGTGATCCTGCG
tciKKO dwn rev	agctatgaccatgattacgaattcgagctcggtaccCCTCCACCTGCAGCATCTGC
tciKKO2 up rev	attcctcgctggctcaaaGCGAACGGGTGGTCAGCACC
tciKKO2 dwn fwd	accacccgttcgctttgaGCCAGCGAGGAATCAGCATGATG
tciKKO2 dwn rev	agctatgaccatgattacgaattcgagctcggtaccGTCACGGCAGAGCGTGAACC
tciRKO up fwd	caagcttgcatgcctgcaggtcgactctagaggatcGTACGACAGCGACGAGCACTTC
tciRKO up rev	gcttgagcaggtcgactcattaaGGTTCGTCGTCGACCAGTAACAG
tciRKO dwn fwd	ttactggtcgacgacgaaccttaatgaGTCGACCTGCTCAAGCGCATC
tciRKO dwn rev	agctatgaccatgattacgaattcgagctcggtaccGTGTGGCGCAGCGATCACGAC
igiD exp fwd XbaI	tacccggggatcc**tctaga**gtcgacctgcagCCACTCAGAGAGCAAAATGTCCGC
igiD exp rev HindIII	agctatgaccatgattacgcc**aagctt**gcatgGGATGACGACATGCGGCATGG
tciK exp fwd BamHI	tata**ggatcC**ACAAGCTGTCGCAGGAGTTC
tciK exp rev HindIII	tttt**aagctt**GATGCGCTTGAGCAGGTCG
tciR exp fwd BamHI	ccgg**ggatcc**tctagagtcgacctgcagGCCAGCGAGGAATCAGCATGATG
tciR exp rev HindIII	tatgaccatgattacgcc**aagctt**gcatgGTCACGGCAGAGCGTGAACC
nCEN_URA3 fwd	gcccgcttgttcctgaaatgccagtaaagcGTATGGCGTGAAGCGTCCTAAGG
nCEN_URA3 rev	tcagttccggctgggggttcagcagccagcCAGACAAGCTGTGACCGTCTCC
AraC_PBAD fwd	aacgacggccagtgaattcgagctcggtacCTATGCTACTCCGTCAAGCCGTC
AraC_PBAD rev	CTGCAGGTCGACTCTAGAGGATC
rpoN exp fwd	tacccggggatcctctagagtcgacctgcagCAGGTGTACCTGGGCGAACAC
rpoN exp rev	agctatgaccatgattacgccaagcttgcatgGCATGGCGATCTTGTTATGAACAACC
